# Comparing the age and sex trajectories of SARS-CoV-2 morbidity and mortality with other respiratory pathogens

**DOI:** 10.1098/rsos.211498

**Published:** 2022-06-15

**Authors:** C. Jessica E. Metcalf, Juliette Paireau, Megan O'Driscoll, Mathilde Pivette, Bruno Hubert, Isabelle Pontais, Sema Nickbakhsh, Derek A. T. Cummings, Simon Cauchemez, Henrik Salje

**Affiliations:** ^1^ Department of Ecology and Evolutionary Biology, Princeton University, Princeton, NJ, USA; ^2^ Princeton School of Public and International Affairs, Princeton University, Princeton, NJ, USA; ^3^ Mathematical Modelling of Infectious Diseases Unit, Institut Pasteur, CNRS, UMR2000, Paris, France; ^4^ Santé publique France, French national public health agency, Saint Maurice, France; ^5^ Department of Genetics, University of Cambridge, Cambridge, UK; ^6^ Department of Biology, University of Florida, Gainesville, FL, USA; ^7^ MRC–University of Glasgow Centre for Virus Research, Institute of Infection, Immunity and Inflammation, College of Medical, Veterinary and Life Sciences, University of Glasgow, Glasgow, UK

**Keywords:** comparing, age, sex, trajectories, SARS, CoV-2

## Abstract

Comparing age and sex differences in SARS-CoV-2 hospitalization and mortality with MERS-CoV, seasonal coronaviruses, influenza and other health outcomes opens the way to generating hypotheses as to underlying mechanisms driving disease risk. Using 60-year-olds as a reference age group, we find that relative rates of hospitalization and mortality associated with the emergent coronaviruses are lower during childhood and start to increase earlier (around puberty) as compared with influenza and seasonal coronaviruses. The changing distribution of disease risk by age for emerging pathogens appears to broadly track the gradual deterioration of the immune system (immunosenescence), which starts around puberty. By contrast, differences in severe disease risk by age from endemic pathogens are more decoupled from the immune ageing process. Intriguingly, age-specific sex differences in hospitalizations are largely similar across endemic and emerging infections. We discuss potential mechanisms that may be associated with these patterns.

## 1. Introduction

From early in the pandemic, the discrepancy in SARS-CoV-2 outcomes across age was striking; evidence for sex differences followed soon afterwards [1], with male sex identified as a risk factor for severe outcomes [2,3]. Age and sex profiles associated with infectious disease contain the signature of transmission dynamics and vulnerabilities to infection, illness and hospitalization. For example, age groups with higher levels of contact might experience greater incidence of infection [4], while age groups associated with greater frailty (often a characteristic of older individuals) might experience more severe outcomes following infection. The latter is our focus here. Specifically, we seek to characterize patterns of severity of disease following infection across age and sex, and how this differs between emerging versus endemic infections.

Our analysis contrasts three emerging pathogens (SARS-CoV-2, SARS, MERS) with influenza and the endemic coronaviruses. Since our focus is on outcomes following infection, we consider the variability in risk of infection across age (which might be associated with behaviour, susceptibility, etc.) to be secondary to underlying variability associated with severe disease. Put differently, we are assuming that what we observe is a fair reflection of the health outcome of an *infected* individual across different ages and by sex. We then leverage the commonalities and differences of these patterns across age and sex for endemic and emerging pathogens to shed light on the underlying immunological mechanisms associated with the burden of the pandemic virus, SARS-CoV-2.

## 2. Results

**Figure 1.**
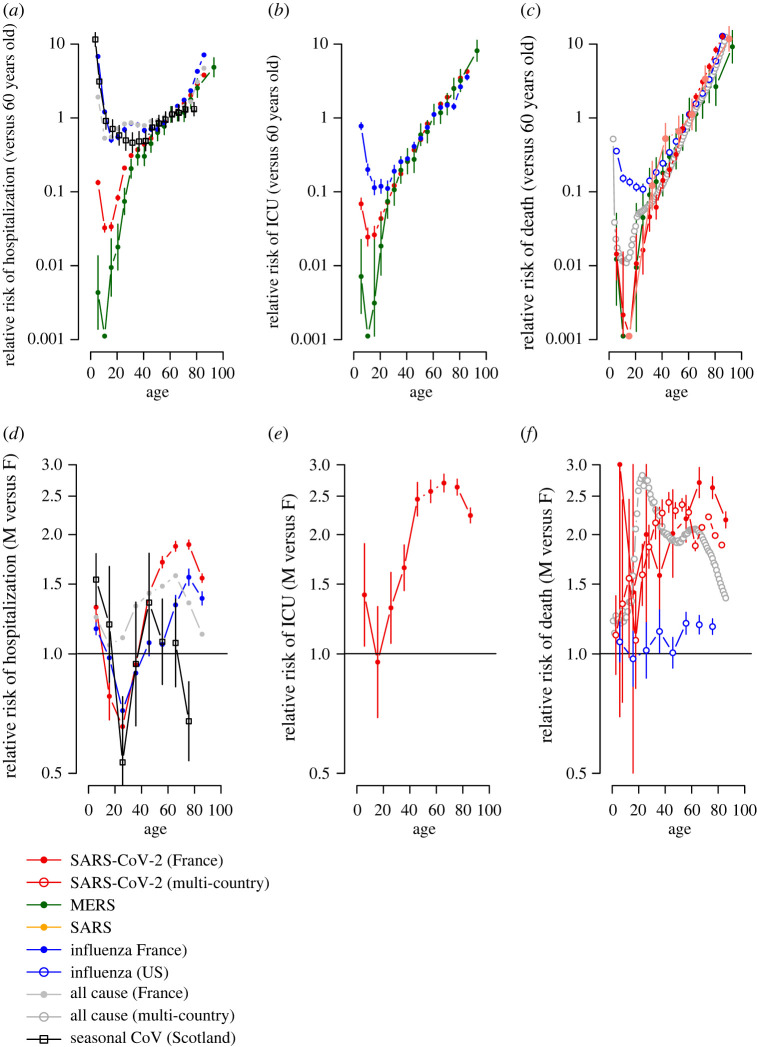
Relative risk of hospitalization, ICU or death for different coronaviruses, influenza as well as underlying levels for all causes. (*a*) Relative incidence of COVID-19 (red), MERS (green), influenza (blue), seasonal coronaviruses (black) hospitalization for each age as compared with 60-year-olds. The relative incidence of all emergency visits is shown as a comparison (grey). (*b*) Relative incidence of COVID-19 (red), MERS (green), influenza (blue) hospitalization requiring ICU for each age as compared with 60-year-olds. (*c*) Relative incidence of COVID-19 (red), MERS (green), influenza (blue), SARS (red) deaths for each age as compared with 60-year-olds. (*d*) Relative incidence of COVID-19 (red), influenza (blue) and seasonal coronaviruses (black) hospitalization for each age comparing males with females. The relative incidence of all emergency visits is shown as a comparison (grey). (*e*) Relative incidence of COVID-19 hospitalization requiring ICU for each age comparing males with females. (*f*) Relative incidence of COVID-19 deaths likewise.

We analysed age and sex data for hospitalization, severe disease (requiring intensive care or resulting in death) and death for SARS-CoV-2 (France and globally), MERS-CoV (Saudi Arabia), SARS (China), influenza (France and USA), as well as hospitalization for seasonal coronaviruses (Scotland), and all-cause hospitalization (France) and mortality (28 European countries) (electronic supplementary material, figure S1). While underlying differences in the (often unknown) underlying population attack rates make comparisons in the absolute risk of hospitalization or death difficult across pathogens, examinations into the patterns of disease by age and sex can provide insight into potential mechanisms driving severe disease. For each pathogen, we compare the incidence of hospitalization and mortality within each age-group with the incidence observed in 60-year-olds (figure 1*a–c*) and fit generalized additive models with spline terms where outcome data is available in single-year age classes (electronic supplementary material, figure S2). This approach also allows us to adjust for differences in mortality between pathogens (and potentially countries) that affect all age groups and the age structure of the underlying population. We also compare the incidence between males and females (figure 1*d–f*; electronic supplementary material, figure S2).

The relative increase in the pathogen-specific probabilities of hospitalization, severe disease and death are strikingly consistent for older individuals across pathogens as well as with all-cause hospitalization and mortality. For COVID-19 cases over 40 years of age, each additional year of life was associated with a 10.7% (95 %CI: 10.3–11.0) increase in probability of death. This was very similar to SARS (10.2%, 5.2–15.5), MERS-CoV (7.1%, 6.2–8.0), influenza (8.5%, 8.1–8.8) and all-cause mortality (10.2%, 10.1–10.4%), and, indeed, the latter has been previously noted [5,6]. Influenza age distributions were also consistent across countries and across different reporting systems within the same country (electronic supplementary material, figures S3 and S4). However, there are notable departures between pathogens among younger age groups.

Qualitatively, all age-patterns of relative risk by age echo the classic U-shape of human mortality: initially high levels of infant hospitalization, severe disease and death first fall, and then increase (figure 1*a–c*; electronic supplementary material, figure S2). However, the age at which the increase in hospitalization, severe disease or mortality occurs differs considerably. In particular, for influenza, seasonal coronaviruses and all-cause hospitalizations, rates remain relatively low between ages 10 and 50, while a stark increase is observed for SARS-CoV-2 right after the minima of 9; and for MERS-CoV from 6 years (figure 1*a*). Severe disease (figure 1*b*) and deaths (figure 1*c*) also exhibit a steeper increase from a younger age for the non-seasonal coronaviruses.

Sex differences in immunity [7,8] provide another lens for evaluating possible mechanisms underpinning the healthcare burden of SARS-CoV-2. In the case of influenza, seasonal coronaviruses and SARS-CoV-2, the burden of hospitalization shifts from being male-biased at the youngest ages, to female-biased between the ages of 18 and 24 and then back toward male again at late ages (figure 1*d*), with a consistent late-life reduction in relative male burden at very late ages presumably attributable to frailty effects (noting small numbers for the seasonal coronaviruses). This broad trajectory is also seen for severe cases of SARS-CoV-2 (figure 1*e*). Mortality data in influenza and SARS-CoV-2 lack these nonlinearities, and the male burden grows consistently relative to the female burden across age, but this increase is much more rapid in SARS-CoV-2 (figure 1*f*).

## 3. Discussion

Commonalities and differences in the severity of outcomes associated with emerging and endemic infections will be rooted in patterns of immune functioning over age and sex. Early in life, development of immune capacity and memory increase protection from infectious diseases. These gains are then eroded by immunosenescence: dysregulation of innate immunity and depletion of adaptive effectors including naive B- and T-cells diminishes defence against infectious pathogens or cancer, and increases inflammation [9]. The latter effect, sometimes termed ‘inflammaging’, is associated with diseases of old age (cardiovascular disease, strokes, diabetes, etc.). The remarkable consistency of late-life increases in hospitalization, ICU and death across pathogens (figure 1*a–c*) suggests a common mechanism, and immunosenescence is the most obvious candidate. But what do discrepancies earlier in life suggest?

Immunopathology-associated cytokines (like IL-6) gradually increase with age in healthy adults [10,11]. Falling levels of protective cytokines (like IL-17A and Ifn-gamma) across paediatric and adult SARS-CoV-2 patients [12] echo this result. Thus, one interpretation of the early start to the steep increase in hospitalization, severe disease and death occurring in younger age groups for SARS-CoV-2 and MERS-CoV than for the endemic pathogens is that, following infection by these pathogens, individuals are more susceptible to the impact of immunosenescence than they would be for endemic infections—so that even the slight changes in immune functioning that start around puberty will lead to worse health outcomes. By contrast, for infections with less sensitivity to immunosenescence, some threshold level of decline in immune functioning might need to be met before worse health outcomes were observed, indicating a later age of onset.

What mechanisms might explain this difference in sensitivity to immunosenescence? The obvious process that affects the emergent coronaviruses differently is the presence of adaptive immunity from prior exposure. Children are likely to experience several influenza infections early in life [13], as well as several seasonal coronaviruses [14] and may accumulate sufficient immunity to buffer them from either infection or its consequences for much of adulthood. This is not the case for the two emergent coronaviruses, SARS-CoV-2 or MERS-CoV, and could explain the differences in the trajectory over adulthood.

Sex differences provide an alternative angle for evaluating the underlying immune drivers of severity of outcomes. Generally, a male bias in risk is expected when weak immune responses underlie significant host damage by a pathogen, while a female bias occurs when strong immune responses promote host damage [7]. SARS-CoV-2 highlights nuances around this broad characterization. Many cytokines and chemokines, and even antibody titres are increased in male patients [8]. Furthermore, whether males or females are more at risk of hospitalization (for SARS-CoV-2 but also other respiratory pathogens) may depend on age (figure 1*d*), with females at greater risk at younger ages.

These observations may be reconciled by noting that increased male immune responses in SARS-CoV-2 might reflect responses to uncontrolled within-host spread; and by considering the effects of sex differences and senescence, some of which are shared across the range of pathogens considered. Chromosomal differences provide greater protection for females both early and late in life. For example, the important pattern-recognition-receptor Toll-like receptor seven sits on the X chromosome and may escape silencing, enabling faster immune detection, and thus lower risk in females [7,8]. At later ages, differences might be amplified by slower female immunosenescence—comparable epigenetic changes happen 5–6 years later in females than males [8]. Lifestyle factors may also play a role: co-morbidities associated with inflammaging but also behaviours like smoking (hypertension, chronic obstructive pulmonary disease) were more common in male patients in an Italian hospital study [15]. Although this may also affect the trajectory of influenza, suggestions of an earlier and more extreme shift of the burden towards males in the case of coronavirus infections (figure 1*d*) is consistent with greater vulnerability linked to immunosenescence. Following puberty, hormonal effects on immunity (e.g. oestrogens are associated with efficient immune recruitment [7] and response could at first be too extreme, yielding initially too high immune responses in females. Indeed, in SARS-CoV-2, female severe outcomes seem driven by over-responding immune systems, i.e. higher levels of innate immune cytokines were associated with worse disease progression in females, but not males [8]. Changing hormone levels, and perhaps the onset of adaptive immunity, at least for influenza and the seasonal coronaviruses, could then diminish this negative effect. Amplification of vulnerability during pregnancy is expected and could increase risk in adult women (although this is generally only up to 5% of the population hospitalized) [16]. Interestingly, the greater risk of influenza or SARS-CoV-2-associated hospitalizations over some part of adulthood in females does not translate into much greater risk of mortality for these pathogens (figure 1*f*), echoing the ‘male–female health survival paradox’, where females in worse health survive better than comparable males, although this effect is typically reported at much later ages. Broader analyses that account for a range of co-morbidities across age and sex alongside immune measures could provide a window into the underlying mechanisms.

Younger individuals have been less vulnerable to severe illness in the SARS-CoV-2 pandemic virus to date. However, comparing the age and sex trajectories of SARS-CoV-2 with other emergent and endemic viruses reveals evidence of a common impact of immunosenescence across pathogens for older individuals but marked departures between pathogens for younger individuals. These findings suggest the impact of the onset of immunosenescence is heightened among populations that have not previously been exposed to a specific pathogen. As the global population ages, the role of immunosenescence in the burden of infectious diseases for which we have little or no pre-existing adaptive immunity may open important vulnerabilities to emerging pathogens.

## 4. Material and methods

### 4.1. Data sources

1. COVID-19 in France. We obtained hospitalization data from the French SI-VIC system from the period March–September 2020. The SI-VIC system is integrated into all hospitals in France and has been used since March 2020 to monitor COVID-19 hospitalized patients. Data are sent daily to Santé publique France, the French national public health agency. We obtained the number of hospitalizations, the number of patients requiring intensive care treatment and the number of patients that died for single year age classes and by sex.

2. Influenza emergency visits in France. We obtained the age and sex distribution of emergency visits from the French OSCOUR network between 2004 and 2019, where influenza was identified as the main or associated clinical diagnosis [17]. The OSCOUR network was set up in 2004 by Santé publique France and now covers about 95% of all French hospitals. We removed data from 2009 from this dataset as 2009 was a pandemic year with different age-distribution of cases.

3. Influenza ICU and death data in France. We obtained the number of cases requiring ICU and the number of deaths by age for the period 2012–2017, from the French national hospital discharge database (PMSI), which contains all records of hospitalizations in all hospitals in France [18]. We included hospital stays where influenza was identified as the main or associated clinical diagnosis.

4. Influenza mortality data USA. We extracted the number of deaths by single year age class and by sex where influenza was the main cause of death for the whole of the USA in 2019 from the US National Vital Statistics System (https://www.cdc.gov/nchs/nvss/deaths.htm).

5. MERS data. We used the MERS database maintained by Andrew Rambaut at the University of Edinburgh that was collated during the MERS outbreak in 2013 (https://github.com/rambaut/MERS-Cases/). This linelist database provides individual age, sex and whether the individual required intensive care treatment and whether they died.

6. SARS mortality data. We obtained the number of SARS deaths in China from Yu *et al*. in the following age classes: 0–24, 25–34, 35–44, 45–54, 55–64, 65–74, 75+ [19].

7. All emergency visits in France. We obtained the age and sex distribution of all coded emergency visits (all causes) in French hospitals from the OSCOUR network between 2004 and 2019.

8. All-cause mortality in 28 European countries. We extracted the all-cause number of deaths by single year age class and by sex for the 28 EU countries (including the UK) through the Euromomo system.

9. Multi-country SARS-CoV-2 mortality model estimates. We obtained estimates of the relative risk of death given infection by age and sex from a previously published model that fitted age and sex-specific COVID-19 death data from 45 countries [1].

10. Seasonal coronavirus hospitalization data. We obtained the age and sex distribution of seasonal coronavirus hospitalization based on diagnostic data for more than 70 000 episodes of respiratory illness in Scotland as described in [14].

### 4.2. Data analyses

1. Comparisons by age

We compared the incidence between individual age groups and the incidence in the age group that contained 60-year-olds. The size of the age group we used depended on the available data. Where the number of counts was available in single-year age groups (SAR-CoV-2 in France, Influenza in France and MERS), we aggregated the data into non-overlapping 5-year age groups. Taking each pathogen in turn, we separately compared the incidence within each age group by dividing the total number of individuals that had the outcome of interest (hospitalization, ICU or death) within an age group with the size of the population in that age group from the national census for that country. We then calculated the relative incidence within each age group as compared with the age group that contained 60-year-olds. This approach allows us to adjust for underlying differences in incidence and outcome across pathogens that affect all age groups in the same way. We considered three different outcomes: hospitalization (or emergency department visit in the case of influenza), severe disease (defined as requiring ICU treatment) and death. For all-cause emergency department visits, we removed the cases that were due to influenza to ensure any patterns were not being driven by influenza admissions. To capture uncertainty we calculated 95% Wald confidence intervals. For the multi-country SARS-CoV-2 mortality estimates, the risk of death given infection was derived from a previously published Bayesian model that combined the results of 22 national-level seroprevalence studies with reported age and sex-specific COVID-19 death data from 45 countries [1].

2. Comparisons between males and females

For all-cause hospitalization and mortality, influenza hospitalization (France) and death (USA), and SARS-CoV-2 hospitalization (France), severe disease (France) and death (multi-country study), sufficient data were available to detect differences by both sex and age. We compared the incidence between males and females using non-overlapping windows of size 10-year age groups (5-year groups in the case of the multi-country SARS-CoV-2 analysis). Taking each pathogen in turn, for each window, we divided the incidence in males with that observed in females. We calculated 95% Wald confidence intervals.

3. Generalized additive models

In the datasets where we had case counts in single year age classes (SAR-CoV-2 in France, Influenza in France and MERS), we also fit generalized additive models using the *mgcv* package in R(Wood 2006) [20]. This approach uses penalized likelihood estimation to fit spline terms. We fit the overall number of cases within single-year age classes using Poisson regression with the log(population size) as an offset. For the SARS-CoV-2 and influenza datasets, we separately fit the counts for males and females. We then calculated the ratio of the predicted incidence between males and females.

## Data Availability

The datasets supporting this article have been uploaded as part of the electronic supplementary material [21].
